# Apparent Optimal Dietary Protein Level and Growth Response of Grow-Out Hybrid Grouper (*Epinephelus akaara* ♀ × *E. lanceolatus* ♂) Under Practical Formulation Conditions

**DOI:** 10.3390/ani16121878

**Published:** 2026-06-17

**Authors:** Taejin Park, Yong Hyun Do, Seong-Mok Jeong, Bo-Hye Nam, Jin Choi, Md Hashibur Rahman, Haham Kim, Seunghyung Lee

**Affiliations:** 1 Aquaculture Research Division, National Institute of Fisheries Science, Busan 46083, Republic of Korea; 2 Aquafeed Research Center, National Institute of Fisheries Science, Pohang 37517, Republic of Korea; 3 Aquaculture Industry Research Division, East Sea Fisheries Research Institute, National Institute of Fisheries Science, Gangneung 25435, Republic of Korea; 4 Major of Aquaculture and Applied Life Sciences, Division of Fisheries Life Sciences, Pukyong National University, Busan 48547, Republic of Korea; hashibur@pukyong.ac.kr (M.H.R.);; 5 Interdisciplinary Program of Marine and Fisheries Sciences and Convergent Technology, Pukyong National University, Busan 48513, Republic of Korea; 6 Feeds and Foods Nutrition Research Center, Pukyong National University, Busan 48547, Republic of Korea

**Keywords:** protein-to-energy ratio, feed utilization, nitrogen-free extract, broken-line regression, plasma antioxidant response

## Abstract

This study evaluated the effects of graded dietary protein levels on growth performance, feed utilization, physiological responses, and antioxidant status in grow-out-stage hybrid grouper (*Epinephelus akaara* ♀ × *E. lanceolatus* ♂) under practical formulation conditions. A total of 450 fish (initial body weight 240 ± 1 g) were randomly assigned to 18 tanks and fed six practical near-isocaloric diets formulated to provide graded crude protein levels of 40% (P40), 45% (P45), 50% (P50), 55% (P55), 60% (P60), or 65% (P65) for 11 weeks. Growth performance indices, including final body weight (FBW), weight gain (WG), specific growth rate (SGR), and feed efficiency (FE), increased from P40 to P60, with the highest values observed in the P60 group. A decline in these indices at P65 indicated reduced nutrient utilization at excessive dietary protein levels within the present diet series. Broken-line regression analysis, using weight gain as the response variable, suggested an apparent optimum around 58–59% crude protein within the present high-fish meal practical formulation matrix, with relatively wide confidence intervals around the breakpoint estimate. Hematological and plasma biochemical parameters, including hematocrit (Hct), hemoglobin (Hb), glucose (GLU), total protein (TP), total cholesterol (TCHO), and blood urea nitrogen (BUN), were not significantly affected by dietary protein level, suggesting stable physiological status across treatments. Dorsal muscle proximate composition and amino acid profiles were similarly unaffected. Antioxidant responses showed moderate variation, with plasma catalase (CAT) activity highest in the P55 group, while plasma glutathione (GSH) levels remained stable across treatments. These findings suggest that, under the present high fish meal practical formulation conditions, an apparent dietary crude protein level of approximately 58–59% may improve growth and feed utilization in grow-out-stage hybrid grouper without markedly altering physiological and antioxidant status.

## Simple Summary

Dietary protein is a key nutrient regulating growth and feed efficiency in carnivorous fish, yet size-specific information for grow-out hybrid grouper remains limited. In this study, grow-out hybrid grouper were fed near-isocaloric practical diets containing graded crude protein levels of 40–65% for 11 weeks. Although the diets were designed to provide graded crude protein levels, they also differed substantially in ingredient composition, carbohydrate/NFE level, and protein-to-energy balance; therefore, these results reflect the overall practical diet formulation, not protein level alone. Growth performance and feed utilization improved up to the P60 diet; however, this response reflected a graded practical diet series formulated primarily through increasing fish meal inclusion and decreasing wheat flour/carbohydrate inclusion. Hematological parameters, plasma biochemical indices, and dorsal muscle composition were largely unaffected across treatments, indicating stable physiological status. Plasma catalase activity, however, varied among groups, suggesting a moderate antioxidant response to the practical diet series. Broken-line regression analysis suggested an apparent optimum around 58–59%, with relatively wide confidence intervals under the present practical formulation conditions. These findings provide size-specific and practically relevant guidance for feed formulation in grow-out hybrid grouper and may help improve feed-use efficiency under commercial rearing conditions.

## 1. Introduction

Groupers (*Epinephelus* spp.) are among the most commercially valuable marine fish in Asia, owing to their high market price, desirable flesh quality, and strong consumer demand [1]. The red-spotted grouper (*Epinephelus akaara*) is particularly valued for its coloration and taste; however, its slow growth rate, requiring nearly three years to reach a market size of approximately 0.6 kg, limits its aquaculture productivity [2,3]. In contrast, the giant grouper (*E. lanceolatus*) exhibits exceptionally rapid growth, reaching 3 kg within one year and over 20 kg within four years [4], but its low tolerance to cold temperatures restricts its cultivation in temperate regions.

To overcome these limitations, a hybrid grouper (*Epinephelus akaara* ♀× *E. lanceolatus* ♂) has been developed, combining the favorable flesh quality and cold tolerance of *E. akaara* with the rapid growth potential of *E. lanceolatus*. Previous studies have demonstrated that this hybrid species exhibits improved growth performance, enhanced feed utilization efficiency, and greater environmental adaptability compared with its maternal parent [5,6,7]. Owing to these advantages, hybrid grouper has emerged as a promising candidate for aquaculture diversification, and increasing research efforts have focused on its physiology [8,9], reproduction and early development [10,11], and nutritional requirements.

Dietary protein is a major nutritional factor influencing growth performance, feed efficiency, metabolism, and health status in carnivorous fish [12]. However, both excessive and insufficient protein supply can impair production efficiency: excessive protein may lead to inefficient nutrient utilization, increased ammonia excretion, and reduced water quality [13], whereas insufficient protein can reduce growth, feed efficiency, and physiological function [14]. Because protein requirements vary according to species, body size, dietary protein quality, protein-to-energy ratio, and feeding strategy, species- and size-specific evaluations remain necessary [15]. Although dietary protein requirements have been investigated in several grouper species, most studies have focused on early juvenile stages, generally reporting optimal protein levels of approximately 48–52% [16,17,18]. Despite the commercial importance of grow-out-stage hybrid grouper, empirical evidence regarding its dietary protein requirements under practical farming conditions remains limited. This limitation is relevant because dietary protein represents a major cost driver in aquafeed formulation, particularly in carnivorous fish diets that continue to rely heavily on fish meal, and optimizing protein utilization is important for both production efficiency and the reduction of nitrogenous waste output [19,20]. Although purified and semi-purified diets are useful for defining theoretical nutrient requirements, practical diets based on conventional feed ingredients provide greater applicability to commercial aquaculture systems [12,21,22]. Because species-specific essential amino acid requirements have not been fully established for hybrid grouper, reference values reported for rainbow trout in NRC [23] were used as a practical benchmark to evaluate dietary essential amino acid adequacy in the present study.

Beyond growth and feed utilization, dietary protein level can influence fish physiology and nutrient deposition patterns. Hematological and biochemical parameters are widely used as indicators of nutritional and physiological status, reflecting metabolic balance and health condition in fish [24]. In grow-out-stage fish, dorsal muscle composition is commonly evaluated as a representative tissue because it directly reflects fillet quality, edible portion yield, and commercial product value [17,25]. It also provides meaningful information on nutrient deposition patterns while minimizing the confounding influence of visceral tissues [26].

In the present study, practical diets were formulated to reflect commercially relevant feed formulation conditions, rather than to isolate the independent effect of dietary protein level. Under such practical formulation conditions, increasing crude protein level necessarily involved changes in ingredient composition, particularly increased fish meal inclusion and reduced wheat flour inclusion. Therefore, the resulting dietary series represented a graded-protein practical formulation matrix, in which crude protein level, fish meal proportion, carbohydrate/NFE level, and protein-to-energy balance changed together. Although this design cannot determine an absolute protein requirement independent of raw-material composition, it provides practical insight into the dietary protein level that supports favorable growth and feed utilization under commercially relevant formulation conditions.

The present study aimed to provide a practical reference point for the growth response of grow-out hybrid grouper under conventional high-quality protein formulation conditions, while recognizing that future studies are needed to reduce fish meal dependence through sustainable replacement strategies. Given these considerations, the present study aimed to evaluate the effects of graded dietary protein levels on growth performance, feed efficiency, hematological responses, dorsal muscle composition, and antioxidant status in grow-out-stage hybrid grouper.

## 2. Materials and Methods

### 2.1. Ethical Approval

The experimental protocol was approved by the Institutional Animal Care and Use Committee of the National Institute of Fisheries Science (Approval No. 2024-NIFS-IACUC-53). All fish were handled in accordance with relevant institutional guidelines to minimize stress.

### 2.2. Preparation of the Experimental Diets

The ingredient composition and proximate analysis of the experimental diets are presented in Table 1. Six near-isocaloric practical diets were formulated to contain graded crude protein levels of 40% (P40), 45% (P45), 50% (P50), 55% (P55), 60% (P60), and 65% (P65). Fish meal was progressively increased as the primary high-quality protein source, whereas wheat flour was decreased inversely as both a carbohydrate source and pellet-binding ingredient. The graded fish meal inclusion was used as a practical formulation approach to achieve increasing crude protein levels while maintaining high protein quality and palatability in this carnivorous marine species. This design was intended to provide a baseline estimate under conventional practical feed conditions, rather than to represent a final sustainable commercial formulation. Consequently, dietary carbohydrate/NFE level and protein-to-energy ratio changed together with crude protein level as part of the practical formulation matrix. Wheat gluten and soybean meal were included as supplementary protein sources, while supplemental fish oil was progressively reduced and soybean oil was slightly adjusted to maintain similar analyzed dietary lipid levels despite the increasing inclusion of fish meal, which contained approximately 8.5% crude lipid. The diets were formulated to be near-isolipidic based on proximate composition; however, digestible energy was not determined. Therefore, the term “near-isocaloric” refers to calculated gross energy values on a dry matter basis, not digestible energy.

For feed preparation, all dry ingredients were weighed and mixed using an electronic industrial mixer (Vertical Blender 12 Inch 20QT VM-20, Hun Woo, Wuhan, China) for 15 min. Approximately 40% filtered tap water was gradually added, followed by an additional 15 min of mixing. Fish oil was then incorporated, and the dough was mixed for a further 10 min. The dough was processed using a pelleting machine (SFD-GT, Shinsung Company, Gimpo-si, Republic of Korea) to produce 2 mm diameter pellets. The pellets were gently broken into smaller pieces by hand, dried in a laboratory drying oven (KE-010 Oven, Dongwon Industries, Seoul, Republic of Korea) at 45 °C for 16 h, sealed in plastic bags, and stored at −20 °C until use. The moisture values reported in Table 1 represented the residual moisture contents of the dried diets measured after pelleting and oven drying, before the start of the feeding trial. Moisture content was not re-analyzed during storage or at the end of the feeding trial. Pellet physical quality and water stability were not quantitatively measured.

The proximate composition of each diet was analyzed in triplicate according to standard AOAC methods [27]. Nitrogen-free extract (NFE) was calculated by difference [100 − (crude protein + crude lipid + ash)] on a dry matter basis. Amino acid profiles of the experimental diets were determined by HPLC (S 433 Amino Acid Analyzer; Sykam GmbH, Eresing, Germany) following acid hydrolysis with 6 N HCl at 110 °C for 24 h, with performic acid oxidation conducted prior to hydrolysis for sulfur-containing amino acids. Amino acid analysis was conducted to evaluate essential amino acid adequacy across the graded practical diet series, rather than to compare different protein feed sources. Although the same major protein ingredients were used across diets, their relative inclusion levels differed substantially, particularly because fish meal inclusion increased progressively with dietary crude protein level. Therefore, amino acid profiles were analyzed to confirm that the graded diet series maintained an acceptable amino acid balance for interpreting growth responses.

The amino acid compositions of the experimental diets are presented in Table 2. The experimental diets were prepared as one batch per treatment; therefore, the nutrient analyses represented analytical replicates rather than independent production replicates. Accordingly, diet composition data were presented descriptively without inferential statistical comparisons. When expressed as a percentage of crude protein, essential amino acid profiles were generally comparable among diets, and all essential amino acid levels met or exceeded the reference requirement values used in the present study. However, apparent amino acid digestibility was not measured; therefore, possible differences in amino acid availability among diets, particularly those associated with increased fish meal inclusion, could not be evaluated directly.

### 2.3. Experimental Conditions

Hybrid grouper were acclimated to laboratory conditions for one week prior to the feeding trial, during which they were fed a commercial diet to minimize handling stress. After acclimation, fish with an initial body weight of 240 ± 1 g (mean ± SE) were randomly distributed into eighteen fiberglass tanks (400 L each), with 25 fish per tank. Each dietary treatment was randomly assigned to three replicate tanks. Seawater was continuously supplied to each tank at a flow rate of approximately 3–5 L/min to ensure adequate water exchange, and dissolved oxygen levels were maintained through continuous aeration using air stones connected to a central blower. Fish were hand-fed the experimental diets to apparent satiation twice daily at 09:00 and 17:00 to minimize feed waste and ensure consistent feed intake (FI) across treatments. During each feeding period, feed was supplied gradually in small portions to apparent satiation, and most pellets were consumed shortly after feed delivery; however, the exact time required for complete feed ingestion was not quantitatively recorded. Water quality was maintained by daily monitoring of feeding behavior and siphoning of feces and uneaten feed from tank bottoms. Mortality was recorded throughout the experimental period, and dead fish were promptly removed upon discovery to prevent deterioration of water quality. 

**Figure 1:**
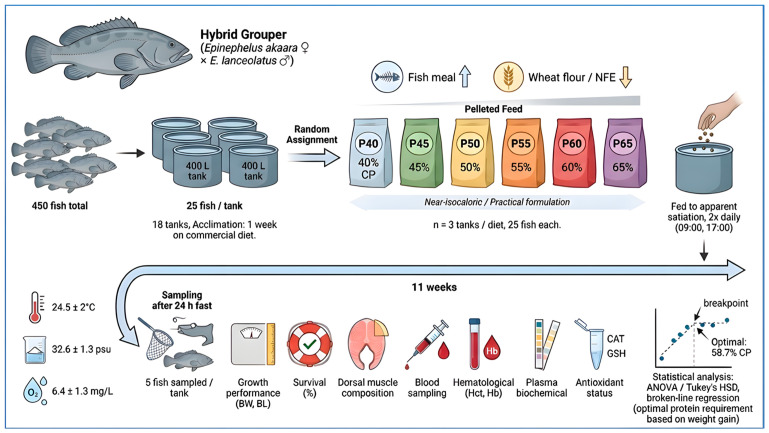
Experimental outline of the 11-week feeding trial evaluating graded dietary protein levels in grow-out hybrid grouper. Fish were assigned to six practical diet groups containing 40–65% crude protein. Growth performance, dorsal muscle composition, hematological and plasma biochemical indices, antioxidant status, and broken-line regression analysis based on weight gain were evaluated.

The rearing system was maintained under a natural photoperiod. Water temperature, salinity, and dissolved oxygen were measured daily using a multiparameter water quality meter (ProQuatro; YSI Incorporation, Yellow Springs, OH, USA). Mean water temperature, dissolved oxygen concentration, and salinity during the 11-week feeding trial were 24.5 ± 2.0 °C, 6.40 ± 1.30 mg/L, and 32.6 ± 1.3 psu, respectively. Nitrogen metabolites and dissolved nutrients, including total ammonia nitrogen, nitrite, nitrate, total nitrogen, and phosphorus, were not measured during the feeding trial. The overall experimental design, including fish allocation, dietary treatments, feeding conditions, sampling procedure, measured parameters, and statistical analysis workflow, is summarized in Figure 1.

### 2.4. Sampling and Blood Analysis

At the end of the 11-week feeding trial, all fish were fasted for 24 h to minimize postprandial variation and ensure accurate physiological measurements. Prior to sampling, fish were anesthetized with 2-phenoxyethanol (200 mg/L; Sigma-Aldrich, Saint Louis, MO, USA) to reduce handling stress. At the time of sampling, the total number and biomass of fish in each tank were recorded to calculate final body weight (FBW), weight gain (WG), specific growth rate (SGR), feed efficiency (FE), protein efficiency ratio (PER), and survival. Following anesthesia, individual body weight and total length were measured using an electronic balance (±0.01 g) and a measuring board (±0.1 cm), respectively, for calculation of the condition factor (CF). Five fish were randomly selected from each tank for further biometric and biochemical analyses. The hepatosomatic index (HSI) and viscerosomatic index (VSI) were determined by dissecting and weighing the liver and visceral tissues of the sampled fish. Dorsal white muscle samples were collected from each fish using sterile scalpels, immediately flash-frozen in liquid nitrogen, and stored at −80 °C until biochemical and amino acid analysis.

Blood samples were collected from the caudal vein using 3 mL heparinized syringes for hematological, plasma biochemical, and antioxidant analyses. Approximately 2 mL of blood was obtained from each fish. Hemoglobin (Hb) concentration was immediately determined using the cyanmethemoglobin method: 20 µL of whole blood was mixed with 5 mL of Drabkin’s reagent (D5941; Sigma-Aldrich, Saint Louis, MO, USA), incubated for 10 min at room temperature, and absorbance was measured at 540 nm using a UV–Vis spectrophotometer (Multiskan GO; Thermo Fisher Scientific, Waltham, MA, USA). Hematocrit (Hct) was determined by centrifuging blood-filled microcapillary tubes using a hematocrit centrifuge (IEC Clinical Centrifuge; International Equipment Corporation, Needham Heights, MA, USA) at 12,000 rpm for 5 min and measuring the packed cell volume using a micro-capillary reader (International Equipment Company, Needham Heights, MA, USA). The remaining blood was centrifuged using a refrigerated centrifuge (Combi 514R; Hanil Scientific Incorporation, Gimpo, Republic of Korea) at 3000× *g* for 10 min at 4 °C to separate plasma, which was immediately placed on ice and stored at −80 °C until analysis.

Plasma biochemical parameters, including GLU, TP, BUN, and TCHO, were analyzed using a FUJI DRI-CHEM NX400i dry chemistry analyzer (FUJIFILM Corporation, Tokyo, Japan), based on dry-slide enzymatic colorimetric assays. Analyte concentrations were determined by automated incubation followed by photometric detection using manufacturer-provided calibration curves. Internal quality control checks and instrument calibration were performed before each analysis in accordance with the manufacturer’s instructions.

Antioxidant biomarkers were analyzed using commercial assay kits (Cayman Chemical Company, Ann Arbor, MI, USA). Plasma GSH concentration was determined using a GSH assay kit based on the colorimetric reaction between reduced glutathione and 5,5′-dithiobis-(2-nitrobenzoic acid) (DTNB), producing a yellow-colored product (5-thio-2-nitrobenzoic acid; TNB) detected at 405 nm using a microplate reader (Synergy HTX; BioTek Instruments, Winooski, VT, USA). Plasma GSH concentrations were calculated from a standard curve and expressed as µmol/mL plasma. Plasma CAT activity was quantified based on the decomposition of hydrogen peroxide (H_2_O_2_): CAT catalyzes the reaction of methanol with residual H_2_O_2_ to generate formaldehyde, which reacts with Purpald^®^ to produce a purple chromophore measured at 540 nm. Plasma CAT activity was expressed as nmol formaldehyde produced min^−1^ mL^−1^ plasma. All samples were analyzed in duplicate under chilled conditions to minimize enzymatic degradation and ensure analytical reliability.

The calculated indices were expressed according to the following formulas:

Weight gain (WG, %) = (FBW − IBW)/IBW × 100;

Specific growth rate (SGR, % day^−1^) = [ln (FBW) − ln (IBW)]/day of feeding × 100;

Feed intake (FI, g/fish) = total dry feed consumed/number of fish;

Feed efficiency (FE, %) = weight gain (g)/feed intake (g) × 100;

Protein efficiency ratio (PER) = weight gain (g)/protein intake (g);

Survival rate (SR, %) = [(number of total fish − number of dead fish)]/Number of total fish × 100;

Hepatosomatic index (HSI, %) = (Liver weight/FBW) × 100;

Viscerosomatic index (VSI, %) = (Viscera weight/FBW) × 100;

Condition factor (CF) = (FBW/Total length^3^) × 100.

The units for weight and length were grams and centimeters, respectively.

### 2.5. Statistical Analysis

All data were expressed as mean ± standard error (SE). Prior to analysis, data were tested for normality and homogeneity of variance using the Shapiro–Wilk and Levene’s tests, respectively. Differences among dietary treatments were assessed using one-way analysis of variance (ANOVA), and Tukey’s HSD test was applied for post hoc comparisons when significant treatment effects were observed. The level of significance was set at *p* < 0.05. All statistical analyses were conducted using SPSS software (version 20.0; IBM Corp., Armonk, NY, USA). The dietary protein requirement was estimated based on weight gain as a response variable using a broken-line (BL) regression model in R version 4.3.0 (R Foundation for Statistical Computing, Vienna, Austria).

The one-slope broken-line model was defined as:

Y = L − U × (R − X) for X < R, and Y = L for X ≥ R,

where Y is the response variable (weight gain), X is dietary protein level, R is the estimated breakpoint, L is the response at the breakpoint, and U is the slope below the breakpoint.

The quadratic broken-line model was defined as:

Y = L − U × (R − X)^2^ for X < R, and Y = L for X ≥ R,

where the parameters are defined as above. Model parameters were estimated in R by nonlinear least-squares regression, and convergence was accepted when parameter estimates stabilized and the residual sum of squares was minimized under the default convergence criteria of the fitting function.

## 3. Results

### 3.1. Growth Performance

The growth performance of hybrid grouper fed six graded dietary protein levels for 11 weeks is summarized in Table 3. Growth performance indices, including FBW (*p* = 0.0026), WG (*p* = 0.0009), SGR (*p* = 0.0016), and FE (*p* = 0.0004), increased progressively as dietary protein increased from 40% to 60%, with the highest values observed in fish fed the P60 diet. When dietary protein was further increased to 65%, these indices declined numerically relative to the P60 group, indicating that further increases in dietary protein did not provide additional growth benefits under the present practical formulation conditions. Survival rate (SR) remained high across all treatments, ranging from 96.0% to 100%, and did not differ significantly among dietary groups. FI did not differ significantly among dietary treatments (*p* = 0.0853), although some numerical variation was observed among diets. Similarly, PER did not differ significantly and remained relatively stable across all dietary groups. No significant differences in VSI, HSI, or CF were observed among dietary treatments.

**Figure 2:**
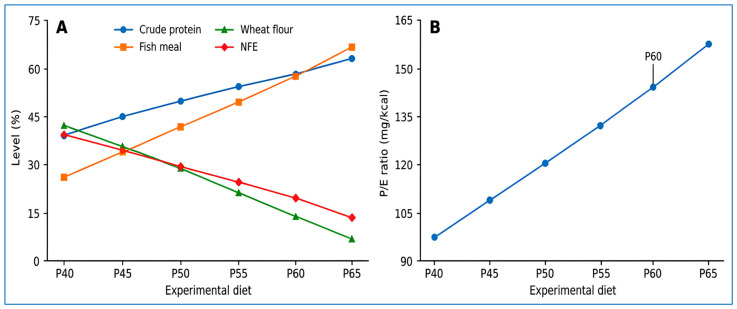
Practical formulation matrix of the experimental diets used for grow-out hybrid grouper. (**A**) Concurrent changes in dietary crude protein, fish meal inclusion, wheat flour inclusion, and nitrogen-free extract (NFE) across the experimental diets (P40–P65); (**B**) progressive increase in crude protein-to-gross energy ratio (P/E ratio) across the same diet series. Together, these variables illustrate that the experimental diets represented a graded practical formulation matrix in which dietary crude protein level co-varied with ingredient composition and protein-to-energy balance.

The formulation changes across the experimental diets are summarized in Figure 2. As dietary crude protein increased from P40 to P65, fish meal inclusion and crude protein-to-gross energy ratio increased progressively, whereas wheat flour inclusion and nitrogen-free extract (NFE) decreased. These trends confirm that the dietary treatments represented a practical formulation matrix rather than an isolated change in crude protein alone.

**Figure 3:**
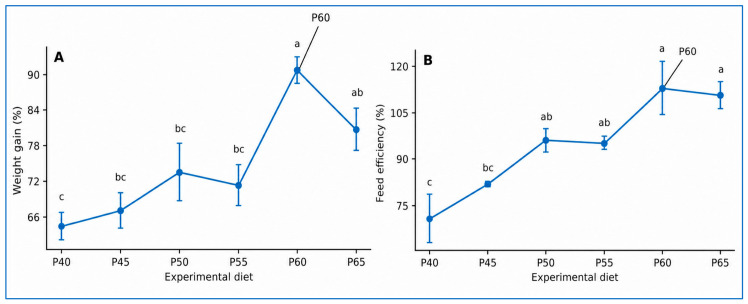
Growth response of grow-out hybrid grouper fed graded practical diets for 11 weeks. (**A**) Weight gain (WG, %); (**B**) feed efficiency (FE, %). Values are presented as mean ± SE (*n* = 3). Different lowercase letters above data points indicate significant differences among dietary treatments (Tukey’s HSD test, *p* < 0.05). The P60 diet showed the most favorable overall growth response under the present practical feeding conditions.

The growth responses associated with the practical diet formulations are summarized in Figure 3. WG increased from P40 to P60, with the highest numerical value observed in the P60 group, and then slightly decreased in the P65 group. WG ranged from 64.5% in P40 to 91.0% in P60, followed by 80.9% in P65. Tukey’s HSD test showed that WG in P60 was significantly higher than that in P40, P45, P50, and P55, but not significantly different from P65. FE showed a similar pattern, with significantly higher values in P60 and P65 than in P40 and P45. Overall, although some statistical overlap was observed between P60 and P65, the highest numerical WG and FE were recorded in fish fed the P60 diet.

**Figure 4:**
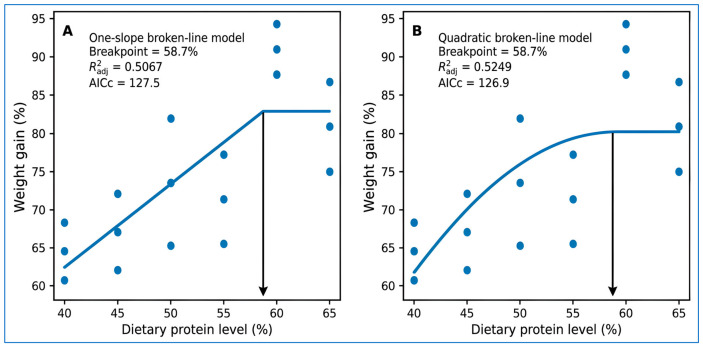
Broken-line regression analysis of weight gain in hybrid grouper fed diets containing graded protein levels for 11 weeks. The (**A**) shows the one-slope broken-line model, and (**B**) shows the quadratic broken-line model. The estimated breakpoint values (± SE) with 95% confidence intervals from the one-slope and quadratic broken-line models were 58.7 ± 4.5% (95% CI: 49.1–68.4%) and 58.7 ± 3.8% (95% CI: 50.7–66.8%) crude protein, respectively. Arrows indicate the estimated breakpoint values, suggesting an apparent optimum around 58–59% CP under the present practical formulation conditions, with relatively wide confidence intervals.

The relationship between WG and dietary protein level, evaluated using broken-line (BL) regression models, is presented in Figure 4. Both the one-slope and quadratic BL models estimated a breakpoint of 58.7% crude protein. The estimated breakpoint values were 58.7 ± 4.5% CP for the one-slope BL model, with a 95% confidence interval of 49.1–68.4%, and 58.7 ± 3.8% CP for the quadratic BL model, with a 95% confidence interval of 50.7–66.8%. The quadratic BL model showed a slightly higher adjusted coefficient of determination (R^2^adj = 0.5249) and a lower corrected Akaike information criterion (AICc = 126.9) than the one-slope BL model (R^2^adj = 0.5067; AICc = 127.5). Therefore, the quadratic BL model was selected to represent the apparent optimal dietary crude protein level under the present practical formulation conditions. However, the modest adjusted R^2^ values and relatively wide confidence intervals indicate considerable uncertainty around the precise breakpoint estimate; therefore, the broken-line models suggested an approximate apparent optimum around 58–59% CP, rather than defining a precise fixed requirement.

### 3.2. Proximate Composition and Amino Acid Profiles of Dorsal Muscle

The proximate composition of dorsal muscle of hybrid grouper fed graded dietary protein levels for 11 weeks is presented in Table 4. No significant differences were observed among treatments for moisture, crude protein, crude lipid, or crude ash contents, indicating that graded dietary protein levels did not markedly alter muscle nutrient deposition under the experimental conditions. The amino acid composition of dorsal muscle is presented in Table 5 as supporting information. Dietary treatment did not significantly affect essential or non-essential amino acid profiles, indicating that amino acid percentage composition was not a major response variable under the present experimental conditions.

### 3.3. Hematological, Plasma Biochemical, and Antioxidant Parameters

The hematological, plasma biochemical, and antioxidant parameters of hybrid grouper fed graded dietary protein levels for 11 weeks are summarized in Table 6.

Hematocrit (Hct) and hemoglobin (Hb) concentrations were not significantly altered by dietary protein level (*p* > 0.05). Plasma biochemical parameters, including GLU, TP, TCHO, and BUN, were similarly unaffected across treatments (*p* > 0.05), indicating stable metabolic and physiological status throughout the experimental period.

**Figure 5:**
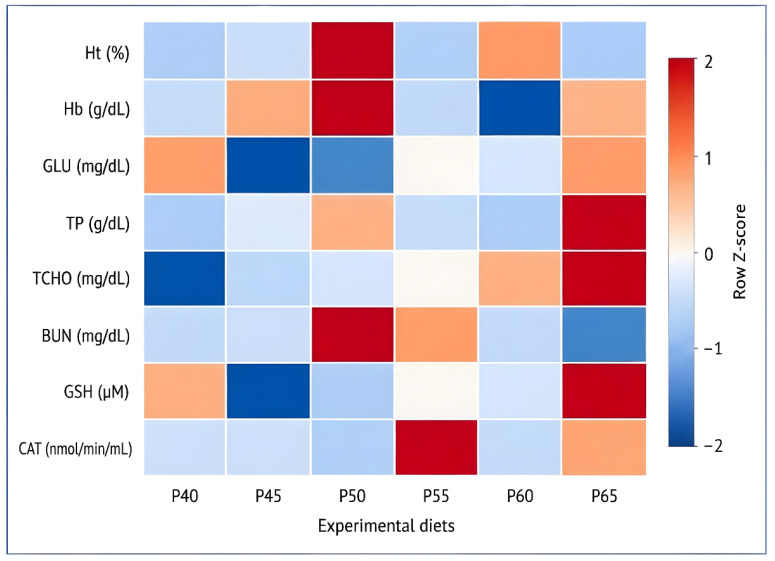
Heatmap summarizing hematological, plasma biochemical, and antioxidant responses of hybrid grouper fed graded dietary protein levels for 11 weeks. Values are presented as row-wise Z-scores of treatment means for hematocrit (Hct), hemoglobin (Hb), glucose (GLU), total protein (TP), total cholesterol (TCHO), blood urea nitrogen (BUN), glutathione (GSH), and catalase (CAT). Red indicates relatively higher values and blue indicates relatively lower values within each parameter. This figure is intended to facilitate visual comparison of relative treatment patterns rather than statistical inference. Among the displayed variables, CAT was the only parameter that differed significantly among treatments (*p* < 0.05).

Among the antioxidant parameters, plasma GSH levels did not differ significantly among treatments (*p* > 0.05). In contrast, plasma CAT activity differed significantly among dietary groups (*p* = 0.0072), with the highest activity observed in the P55 group and the lowest in the P50 group. These results suggest that CAT activity responded selectively to dietary protein level, potentially reflecting variation in protein metabolism and associated oxidative processes in grow-out hybrid grouper. The relative response patterns of hematological, plasma biochemical, and antioxidant parameters across dietary treatments are summarized in Figure 5. The heatmap shows that most parameters remained relatively stable among treatments, whereas CAT exhibited a more distinct treatment-specific pattern.

## 4. Discussion

The present study demonstrated that the graded practical diet series significantly influenced growth performance in grow-out hybrid grouper. WG and SGR increased from P40 to P60 but declined at P65, suggesting that the P60 diet provided the most favorable nutritional balance under the present feeding conditions. The graded increase in dietary crude protein was obtained primarily by increasing fish meal inclusion and reducing wheat flour/NFE level. Therefore, the diets also differed in ingredient composition, carbohydrate/NFE level, lipid-source composition, and protein-to-energy ratio. Accordingly, the observed growth response likely reflected the combined effects of dietary protein level and concurrent formulation changes within a practical feed matrix, rather than the effect of crude protein alone. The estimated value should therefore be regarded as an apparent optimal dietary crude protein level under the present practical formulation conditions, not as an absolute protein requirement independent of raw-material composition. In this practical diet series, the improved growth response from P40 to P60 may have been associated not only with increased crude protein supply, but also with higher fish meal inclusion, improved availability of essential amino acids, and a progressive reduction in dietary carbohydrate/NFE level. Although the same major protein ingredients were used across diets, their relative inclusion levels differed because fish meal increased progressively with dietary crude protein level. Therefore, amino acid analysis was useful for confirming essential amino acid adequacy across the graded practical diet series. When expressed relative to crude protein, the analyzed essential amino acid profiles were generally comparable among diets. However, because apparent amino acid digestibility was not measured, possible differences in amino acid availability could not be directly evaluated. In addition, because carnivorous marine fish generally have limited capacity to utilize high carbohydrate levels, the reduction in wheat flour/NFE may have contributed to improved nutrient utilization in the higher-protein diets. The increase in protein-to-energy ratio up to P60 may also have provided a more favorable balance between protein supply and non-protein energy for somatic growth. However, the further increase in protein-to-energy ratio in P65 may have exceeded the level efficiently utilized for growth, leading to greater amino acid catabolism and no additional growth benefit. Therefore, the apparent optimum around 58–59% CP likely reflects the combined influence of dietary protein level, fish meal inclusion, reduced carbohydrate/NFE, and protein-to-energy balance within the present practical formulation matrix. Previous studies have reported that excessive dietary protein impairs nutrient utilization and feed efficiency, ultimately reducing growth in grouper [28]. Similar decreases in WG and SGR at protein levels exceeding the optimal range have been reported in Alaska pollock (*Gadus chalcogrammus*) [29], common two-banded seabream (*Diplodus vulgaris*) [30], rainbow trout (*Oncorhynchus mykiss*) [31], olive flounder (*Paralichthys olivaceus*) [32], flathead grey mullet (*Mugil cephalus*) [33], and Nile tilapia (*Oreochromis niloticus*) [34]. Fish expend substantial metabolic energy to catabolize excess dietary protein, thereby reducing energy available for somatic growth and negatively affecting overall growth performance [15].

Lee et al. [35] reported that, in broken-line (BL) regression models, a higher coefficient of determination (R^2^) and a lower Akaike information criterion (AICc) indicate better model fit. In the present study, the quadratic BL model showed a slightly higher adjusted R^2^ and lower AICc than the one-slope BL model. Therefore, the quadratic BL model was used to represent the apparent optimal dietary crude protein level. The broken-line regression models suggested an apparent optimum around 58–59% CP for grow-out hybrid grouper under the present practical formulation conditions. This value is higher than the optimum protein level reported for juvenile orange-spotted grouper, *E. coioides* (52.2%) [36], *E. lanceolatus* (56%) [37], *E. malabaricus* (55%) [38], *E. fuscoguttatus* (50%) [17], *E. moara* (54.6%) [26], and the hybrid *E. fuscoguttatus* ♀ × *E. lanceolatus* ♂ (53.5%) [25]. However, as shown in the results, the adjusted R^2^ values were modest and the 95% confidence intervals for the breakpoint were relatively wide. Therefore, the estimated value should be regarded as an approximate apparent optimum within the present high-fish meal practical formulation matrix, rather than as a precise or universal protein requirement.

The apparent optimum estimated in the present study was higher than values typically reported for smaller grouper juveniles, which generally range around 50% [16,17]. This difference may be associated with the larger fish size, fast-growing hybrid genotype, and practical diet series used in the present study, in which crude protein level varied concurrently with ingredient composition and protein-to-energy balance. Differences in diet type, protein sources, non-protein energy levels, amino acid composition, rearing conditions, and regression models among studies may also have contributed to this variation [21,35,39,40]. In the present study, crude protein increased while gross energy decreased slightly, resulting in a progressive increase in the crude protein-to-gross energy ratio from 95.5 mg/kcal in P40 to 158.4 mg/kcal in P65. The superior growth and feed efficiency observed in the P60 group suggest that approximately 58–60% dietary protein, corresponding to a crude protein-to-gross energy ratio of about 145 mg/kcal, provided a favorable balance between protein supply and dietary energy under the present feeding conditions. In contrast, the reduced growth response in P65 may indicate that further increases in protein supply exceeded the level efficiently utilized for growth.

Feed efficiency (FE) and protein efficiency ratio (PER) were lower in the P65 group than in the P60 group, suggesting reduced protein utilization at excessive dietary protein levels. Similar to previous findings [41], this may be attributed to the increased energetic cost of metabolizing surplus protein. Excess amino acids may be diverted to energy production or converted to glucose and lipids [21,42]. In contrast, fish fed the P40 diet showed the lowest WG, SGR, and FE, indicating insufficient amino acid supply for tissue synthesis and metabolism [43]. Although feed intake (FI) did not differ significantly among treatments, minor numerical differences were observed, which may reflect normal variation in voluntary feed intake under practical feeding conditions [44]. Therefore, FI was interpreted descriptively and was not considered a primary driver of the significant growth and feed-efficiency responses observed in this study. Although the experimental diets were formulated within a narrow gross energy range, the graded protein levels were obtained through concurrent adjustments in fish meal, wheat flour, and lipid sources. This further supports the interpretation that the growth responses reflected the overall practical formulation matrix rather than isolated effects of crude protein alone. Thus, within the present practical formulation matrix, the improved performance in the higher-protein diets may reflect a more favorable balance between protein supply and carbohydrate/NFE reduction, rather than superior protein utilization alone.

Excess dietary carbohydrates have been reported to impair growth and promote hepatic and visceral energy deposition in fish [45,46]. Although HSI and VSI did not differ significantly among treatments in the present study, both indices showed lower numerical values in the higher-protein diets, in which wheat flour/NFE levels were reduced. These numerical patterns may reflect differences in energy deposition associated with dietary protein and carbohydrate balance [47]. Therefore, some contribution of dietary carbohydrate level to the observed growth responses cannot be excluded. The progressive reduction in wheat flour inclusion may also have influenced feed-processing characteristics. This is because wheat flour can contribute to dough cohesion, pellet formation, and pellet integrity. However, because pellet physical quality and water stability were not quantitatively evaluated, possible processing-related effects associated with reduced wheat flour inclusion should be taken into account when interpreting the present findings, as further addressed in the limitations section.

Most previous studies assessing body composition in groupers have focused on whole-body analysis in small juveniles [17]. In larger or grow-out-stage fish, dorsal muscle composition is commonly evaluated as a representative tissue because of its direct relevance to fillet quality and commercial value [25]. Assessing dorsal muscle composition provides meaningful information on nutrient deposition patterns while minimizing the confounding influence of visceral tissues, particularly lipid-rich organs [26]. In the present study, the absence of significant differences in dorsal muscle proximate composition suggests that graded dietary protein levels did not markedly alter muscle nutrient deposition under the experimental conditions. Although dorsal muscle crude lipid did not differ significantly among treatments, a decreasing trend was observed as dietary protein level increased, with the lowest numerical value observed in the P65 group. This pattern should be interpreted cautiously because the differences were not statistically significant. Nevertheless, the low numerical muscle lipid content in the P65 group may be related to concurrent formulation changes across the diet series. First, fish oil inclusion decreased from 5.90% in P40 to 1.40% in P65, which may have reduced the direct contribution of dietary lipid to muscle lipid deposition. Second, the progressive reduction in wheat flour/NFE level may have limited carbohydrate-derived substrate availability for de novo lipogenesis. Third, the P65 diet had the highest protein-to-energy ratio, and excessive dietary protein may increase amino acid catabolism and metabolic energy expenditure, thereby reducing energy available for lipid deposition. Finally, in the higher-protein diets, available nutrients may have been preferentially partitioned toward protein metabolism and somatic growth rather than lipid accumulation in muscle tissue. Because dietary digestibility was not assessed in the present study, it is not possible to directly quantify whether energy supply was adequate across all treatments; this represents a limitation that future studies should address through apparent digestibility coefficient determination. Because the amino acid profiles were expressed as a percentage of crude protein and showed only limited variation among treatments, these data should be interpreted mainly as supporting information for diet and muscle characterization rather than as a central biological response. The primary responses in the present study were the improvements in growth performance and feed utilization observed up to the P60 diet.

Hematological parameters are widely recognized as indicators of fish health and physiological condition [24]. In the present study, hematological and plasma biochemical indices, including Hct, Hb, GLU, TP, TCHO, and BUN, were not significantly affected by dietary protein level, indicating that dietary protein level within the tested range did not adversely influence the physiological status of hybrid grouper. Similar findings have been reported in rainbow trout and Nile tilapia, where blood parameters remained stable when dietary protein levels met nutritional requirements [24,31]. The limited variation in these physiological indicators suggests that the tested dietary protein range did not impose marked metabolic disturbance under the 11-week feeding conditions. However, unchanged routine plasma biochemical indices do not exclude the possibility of subtler or longer-term metabolic effects, particularly under extended grow-out conditions or additional environmental stressors.

Fish are known to regulate antioxidant enzyme systems to mitigate oxidative stress and protect against cellular damage, including lipid peroxidation, enzyme inactivation, and nucleic acid degradation [48]. In the present study, plasma GSH levels were not significantly influenced by dietary protein level. In contrast, plasma CAT activity differed significantly among treatments, with the highest activity observed in the P55 group. Similar responses have been reported in other fish species, where moderate dietary protein levels enhanced antioxidant enzyme activities without inducing oxidative stress [25,37]. These results suggest that basal antioxidant status remained largely stable across treatments, while plasma CAT activity responded dynamically to changes in protein metabolism. This pattern likely reflects the balance between nutrient utilization efficiency and oxidative processes in grow-out hybrid grouper. The significant CAT response, together with stable GSH levels, suggests a moderate and selective antioxidant adjustment rather than broad oxidative stress. Because the present trial lasted 11 weeks and only plasma CAT and GSH were measured, longer feeding trials combined with tissue-level antioxidant enzymes and lipid peroxidation markers may be required to determine whether dietary protein level induces longer-term oxidative effects. Therefore, the antioxidant responses observed in the present study should be regarded as preliminary indicators of oxidative status. Future studies incorporating additional markers, such as superoxide dismutase (SOD), glutathione peroxidase (GPx), and lipid peroxidation indices, together with tissue-level analyses, would provide a more comprehensive understanding of oxidative responses to dietary protein level.

### Limitations and Future Directions

The findings of the present study should be considered in the context of several methodological limitations. First, the use of practical diets limited the ability to separate the effects of dietary crude protein level from concurrent changes in ingredient composition, including fish meal, wheat flour, carbohydrate/NFE level, lipid sources, and protein-to-energy ratio. Second, apparent digestibility coefficients were not determined; therefore, the efficiency of dietary protein, lipid, carbohydrate, and energy utilization could not be directly quantified. Third, nitrogenous waste and dissolved nutrient concentrations, including total ammonia nitrogen, nitrite, nitrate, total nitrogen, and phosphorus, were not measured during the feeding trial. Fourth, pellet physical quality and water stability were not evaluated; therefore, possible effects of pellet durability, production of fines, or nutrient leaching should be taken into account when interpreting the present findings. Finally, fatty acid profiles were not analyzed, although fish oil inclusion decreased and soybean oil inclusion increased across the diet series. From a sustainability perspective, the high fish meal inclusion required to achieve the apparent optimum in the present formulation matrix should be regarded as a limitation rather than a recommendation for unrestricted fish meal use. The present study provides a nutritional reference point under conventional high-quality protein conditions, but future studies should determine whether the same growth response can be maintained using lower fish meal levels through balanced amino acid supplementation, alternative protein sources, and optimized protein-to-energy ratios. Future studies should include digestibility measurements, nitrogenous waste monitoring, standardized pellet-quality assessments, nutrient-leaching tests, and dietary and tissue fatty acid analyses to better distinguish the effects of dietary protein level from those of ingredient composition and feed-processing characteristics. From an economic and sustainability perspective, the recommendation of approximately 58–60% dietary protein should be considered formulation-specific, as this level was obtained within a high-fish meal diet matrix. Although the P60 diet supported the most favorable growth and feed-utilization responses, further increases in protein and fish meal inclusion did not provide additional growth benefits, indicating that excessive protein inclusion may reduce economic efficiency. Therefore, future feed development for grow-out hybrid grouper should evaluate strategies to maintain the growth performance observed at the apparent optimum while reducing reliance on fish meal through balanced protein energy formulation and partial replacement with sustainable alternative protein sources. In such future studies, dietary and muscle amino acid profiles, apparent amino acid digestibility, hematological parameters, plasma biochemical indices, and antioxidant biomarkers should also be evaluated to determine whether alternative protein formulations can support both growth performance and physiological health. In addition, metabolomics-based analyses would be useful to identify diet-induced metabolic changes and to provide mechanistic insight into how different protein sources or formulation matrices affect nutrient utilization, energy metabolism, and physiological responses. Because many physiological, biochemical, antioxidant, proximate composition, and amino acid parameters did not differ significantly among treatments, the present study should be interpreted primarily as a practical growth-response trial rather than a mechanistic study. Future trials should use different experimental designs that independently manipulate dietary protein level, fish meal inclusion, carbohydrate/NFE level, and protein-to-energy ratio to clarify the specific factors driving growth and nutrient-utilization responses.

## 5. Conclusions

This study provides the first size-specific estimate of the apparent optimal dietary protein level for grow-out-stage hybrid grouper (*Epinephelus akaara* ♀ × *E. lanceolatus* ♂) at a body weight of approximately 240 g under practical feeding conditions. The graded practical diet series significantly influenced growth performance and feed utilization, with weight gain, specific growth rate, and feed efficiency improving progressively from 40% to 60% dietary protein. However, because increasing crude protein was accompanied by progressive increases in fish meal inclusion and protein-to-energy ratio, together with decreases in wheat flour and carbohydrate/NFE levels, these responses should be interpreted within a high-fish meal practical formulation matrix rather than as the effect of protein alone. The most favorable growth performance was observed in fish fed the P60 diet. The reduced response in the P65 group suggests that further increasing dietary crude protein within this formulation matrix did not provide additional growth benefits. Based on weight gain, the broken-line regression models suggested an apparent optimum around 58–59% CP within the present high-fish meal practical formulation matrix, rather than a precise or absolute protein requirement independent of ingredient composition. Hematological parameters, plasma biochemical indices, and dorsal muscle composition remained largely unaffected across dietary treatments, suggesting stable physiological and metabolic status throughout the experimental period. Among the antioxidant parameters, plasma CAT activity varied significantly among treatments, with peak activity at the intermediate protein level (P55), while plasma glutathione (GSH) levels remained stable, indicating that antioxidant responses were selectively modulated by dietary protein supply.

These findings suggest that, within the present high-fish meal practical formulation matrix, a dietary protein level of approximately 58–60% supported the most favorable growth and feed-utilization responses in grow-out-stage hybrid grouper. The results further highlight that excessive protein inclusion confers no additional growth benefit and may reduce feed efficiency, emphasizing the importance of balanced protein energy formulation for improved economic viability. It should be noted, however, that the optimal diet identified in this study relies on a high fish meal inclusion, and the diets also differed in fish oil composition, which may have independently influenced palatability and nutrient utilization in this carnivorous species. Future studies should therefore evaluate the extent to which fish meal and fish oil can be partially replaced with alternative ingredients while maintaining comparable growth performance, as a step toward more resource-efficient hybrid grouper aquaculture.

## Figures and Tables

**Table 1:** Ingredient and proximate composition of the experimental diets (*).

Ingredient (%)	Experimental Diets					
	P40	P45	P50	P55	P60	P65
Fish meal ^1^	26.5	34.5	42.5	50.5	58.5	66.5
Soybean meal	10.0	10.0	10.0	10.0	10.0	10.0
Wheat gluten	10.0	10.0	10.0	10.0	10.0	10.0
Wheat flour	43.6	36.3	29.0	21.7	14.4	7.10
Fish oil	5.90	5.00	4.10	3.20	2.30	1.40
Soybean oil	0.00	0.20	0.40	0.60	0.80	1.00
Vitamin premix ^2^	1.50	1.50	1.50	1.50	1.50	1.50
Vitamin C	0.50	0.50	0.50	0.50	0.50	0.50
Choline	0.50	0.50	0.50	0.50	0.50	0.50
Mineral premix ^3^	1.50	1.50	1.50	1.50	1.50	1.50
Proximate composition (% of dry matter basis)						
Moisture ^4^	4.08	4.36	5.61	4.45	4.79	4.48
Crude protein	40.3	45.0	49.7	54.4	58.7	63.3
Crude lipid	10.7	10.7	10.6	10.7	10.5	10.5
Ash	7.84	8.92	10.1	10.9	12.1	13.2
NFE ^5^	41.2	35.4	29.6	24.0	18.7	13.0
Gross Energy (kcal/kg DM)	4221.4	4178.2	4126.0	4099.0	4041.0	3997.0
P/E ratio (mg/kcal) ^6^	95.5	107.7	120.5	132.7	145.3	158.4

^1^ Fish meal was composed of a mixture of Chilean sardine (*Strangomera bentincki*), anchovy (*Engraulis ringens*), and jack mackerel (*Trachurus murphyi*), and contained approximately 8.5% crude lipid according to the supplier’s specification. ^2^ Vitamin premix contained the following amount which were diluted in cellulose (g/kg mix): L-ascorbic acid, 121.2; DL-α-tocopheryl acetate, 18.8; thiamin hydrochloride, 2.7; riboflavin, 9.1; pyridoxine hydrochloride, 1.8; niacin, 36.4; Ca-D-pantothenate, 12.7; myo-inositol, 181.8; D-biotin, 0.27; folic acid (98%), 0.68; p-aminobenzoic acid, 18.2; menadione, 1.8; retinyl acetate, 0.73; cholecalciferol, 0.003; cyanocobalamin, 0.003. ^3^ Mineral premix contained the following ingredients (g/kg mix): NaCl, 43.3; MgSO_4_ · 7H_2_O, 136.5; NaH_2_PO_4_ · 2H_2_O, 86.9; KH_2_PO_4_, 239; CaHPO_4_, 135.3; Ferriccitrate, 29.6; ZnSO_4_ · 7H_2_O, 21.9; Ca-lactate, 304; CuCl, 0.2; AlCl_3_ · 6H_2_O, 0.15; KI, 0.15; MnSO_4_ · H_2_O, 2.0; CoCl_2_ · 6H_2_O, 1.0. * Essential amino acid requirements were met through ingredient composition without crystalline amino acid supplementation; amino acid profiles of experimental diets are provided in Table 2. ^4^ Moisture values represent the final analyzed residual moisture contents of the dried diets after pelleting and oven drying. ^5^ Nitrogen-free extract (NFE) was calculated by difference [100 − (crude protein + crude lipid + ash)] on a dry matter basis. Because crude fiber was not separately analyzed, this value represents estimated carbohydrate by difference, including crude fiber. ^6^ Crude protein-to-gross energy ratio (P/E), was calculated as dietary crude protein content (mg/kg diet, dry matter basis) divided by gross energy content (kcal/kg diet, dry matter basis).

**Table 2:** Amino acid profiles of the experimental diets (% of crude protein).

Amino Acids	Experimental Diets						Requirement ^1^
	P40	P45	P50	P55	P60	P65	
Essential amino acids							
Arginine	5.10	5.38	5.29	5.44	5.53	5.72	4.1
Histidine	2.28	2.31	2.28	2.33	2.33	2.35	1.1
Isoleucine	3.91	3.94	3.99	4.10	4.07	4.23	2.2
Leucine	7.09	7.02	7.20	7.35	7.25	7.41	5.8
Lysine	5.40	5.86	6.12	6.50	6.59	6.99	6.1
Methionine	1.82	1.94	2.06	2.14	2.09	2.14	1.3
Phenylalanine	4.14	4.14	4.26	4.15	4.02	4.24	2.0
Threonine	3.54	3.70	3.77	3.89	3.85	4.03	2.6
Valine	4.80	4.81	4.85	4.91	4.89	5.05	2.6
Non-essential amino acids							
Alanine	4.81	5.02	5.26	5.41	5.42	5.64	
Aspartic	7.37	7.68	7.88	8.27	8.22	8.56	
Cystine	0.99	0.96	0.91	0.82	0.82	0.72	
Glutamic	21.6	20.8	19.2	18.9	17.9	17.8	
Glycine	5.12	5.26	5.49	5.65	5.69	5.95	
Proline	6.02	5.62	6.13	5.65	4.85	5.23	
Serine	4.31	4.33	4.26	4.27	4.15	4.22	
Tyrosine	2.68	2.82	2.92	2.92	2.88	2.90	

^1^ Requirement values are based on rainbow trout (*O. mykiss*) as reported in Nutrient Requirements of Fish and Shrimp [22].

**Table 3:** Growth performance of hybrid grouper fed the experimental diets at the end of the 11-week feeding trial.

Parameters	Experimental Diets						*p*-Value
	P40	P45	P50	P55	P60	P65	
IBW ^1^	240 ± 2	239 ± 0	241 ± 2	239 ± 3	240 ± 0	240 ± 1	0.9678
FBW ^2^	394 ± 5 ^b^	399 ± 7 ^b^	418 ± 15 ^ab^	410 ± 8 ^b^	457 ± 4 ^a^	434 ± 10 ^ab^	0.0026
SR ^3^	98.7 ± 1.3	98.7 ± 1.3	98.7 ± 1.3	100 ± 0	96.0 ± 4.0	98.7 ± 1.3	0.8082
WG ^4^	64.5 ± 2.2 ^c^	67.1 ± 2.9 ^bc^	73.6 ± 4.8 ^bc^	71.4 ± 3.4 ^bc^	91.0 ± 1.9 ^a^	80.9 ± 3.4 ^ab^	0.0009
SGR ^5^	0.65 ± 0.02 ^c^	0.67 ± 0.02 ^bc^	0.72 ± 0.04 ^bc^	0.70 ± 0.03 ^bc^	0.84 ± 0.01 ^a^	0.77 ± 0.02 ^ab^	0.0016
FI ^6^	221 ± 20	197 ± 7	184 ± 10	177 ± 8	195 ± 6	176 ± 1	0.0853
FE ^7^	71.2 ± 7.8 ^c^	82.6 ± 0.7 ^bc^	97.2 ± 3.5 ^ab^	96.4 ± 1.7 ^ab^	114 ± 8 ^a^	112 ± 4 ^a^	0.0004
PER ^8^	1.77 ± 0.19	1.81 ± 0.01	1.94 ± 0.06	1.77 ± 0.03	1.91 ± 0.10	1.74 ± 0.07	0.6287
HIS ^9^	2.00 ± 0.02	1.67 ± 0.09	1.73 ± 0.24	1.40 ± 0.15	1.49 ± 0.11	1.32 ± 0.24	0.1044
VSI ^10^	7.03 ± 0.13	6.98 ± 0.30	6.33 ± 0.28	5.77 ± 0.31	6.19 ± 0.24	5.71 ± 0.73	0.1164
CF ^11^	1.75 ± 0.07	1.71 ± 0.07	1.81 ± 0.05	1.78 ± 0.09	1.77 ± 0.06	1.72 ± 0.01	0.8603

Values are means of triplicate groups and presented as mean ± SE (*n* = 3). Values in the same row sharing the same superscript letter are not significantly different (*p* > 0.05), as determined by Tukey’s HSD test. Rows without superscript letters were not significantly different among treatments (*p* > 0.05). ^1^ Initial body weight; ^2^ final body weight; ^3^ survival rate; ^4^ weight gain; ^5^ specific growth rate; ^6^ feed intake; ^7^ feed efficiency; ^8^ protein efficiency ratio; ^9^ hepatosomatic index; ^10^ viscerosomatic index; ^11^ condition factor.

**Table 4:** Proximate composition (%) of the dorsal muscle of hybrid grouper fed the experimental diets at the end of the 11-week feeding trial.

Parameters	Experimental Diets						*p*-Value
	P40	P45	P50	P55	P60	P65	
Moisture	74.6 ± 0.4	74.2 ± 0.2	75.1 ± 0.5	74.7 ± 0.4	75.1 ± 0.8	75.5 ± 0.3	0.4592
Crude protein	20.6 ± 0.3	21.3 ± 0.2	21.3 ± 0.4	21.1 ± 0.4	21.0 ± 0.4	21.6 ± 0.3	0.4246
Crude lipid	3.18 ± 0.33	3.35 ± 1.67	2.33 ± 0.62	3.45 ± 1.42	2.42 ± 0.61	1.59 ± 0.52	0.7481
Crude ash	1.22 ± 0.03	1.29 ± 0.05	1.26 ± 0.03	1.35 ± 0.05	1.34 ± 0.07	1.28 ± 0.04	0.2533

Values are means of triplicate groups and presented as mean ± SE (*n* = 3). Rows without superscript letters were not significantly different among treatments (*p* > 0.05).

**Table 5:** Amino acid profiles of the dorsal muscle of hybrid grouper fed the experimental diets at the end of the 11-week feeding trial (% of crude protein).

Amino Acids	Experimental Diets						*p*-Value
	P40	P45	P50	P55	P60	P65	
Essential amino acids							
Arginine	5.90 ± 0.06	5.90 ± 0.05	5.96 ± 0.02	5.96 ± 0.04	5.98 ± 0.03	5.99 ± 0.05	0.5781
Histidine	2.18 ± 0.03	2.22 ± 0.01	2.24 ± 0.03	2.20 ± 0.02	2.24 ± 0.03	2.22 ± 0.01	0.5072
Isoleucine	4.72 ± 0.05	4.66 ± 0.02	4.74 ± 0.07	4.67 ± 0.05	4.68 ± 0.03	4.70 ± 0.06	0.9012
Leucine	8.00 ± 0.04	8.03 ± 0.05	8.13 ± 0.07	8.02 ± 0.05	8.02 ± 0.02	7.99 ± 0.04	0.3426
Lysine	9.50 ± 0.01	9.58 ± 0.04	9.64 ± 0.03	9.50 ± 0.04	9.48 ± 0.04	9.50 ± 0.06	0.0897
Methionine	2.79 ± 0.02	2.81 ± 0.02	2.72 ± 0.05	2.83 ± 0.02	2.73 ± 0.04	2.86 ± 0.04	0.1131
Phenylalanine	4.05 ± 0.06	4.06 ± 0.02	4.08 ± 0.05	4.02 ± 0.06	4.02 ± 0.04	4.02 ± 0.07	0.9399
Threonine	4.45 ± 0.02	4.43 ± 0.03	4.44 ± 0.02	4.47 ± 0.01	4.48 ± 0.01	4.46 ± 0.02	0.5227
Valine	4.99 ± 0.05	5.01 ± 0.02	5.03 ± 0.08	4.97 ± 0.03	5.0 ± 0.06	4.97 ± 0.02	0.8888
Non-essential amino acids							
Alanine	6.05 ± 0.07	6.04 ± 0.08	6.01 ± 0.04	6.11 ± 0.05	6.03 ± 0.01	6.04 ± 0.02	0.8127
Aspartic	10.3 ± 0.1	10.4 ± 0.1	10.3 ± 0.0	10.3 ± 0.1	10.3 ± 0.1	10.4 ± 0.1	0.9106
Cystine	0.96 ± 0.18	1.02 ± 0.11	1.01 ± 0.14	0.82 ± 0.11	0.95 ± 0.12	0.77 ± 0.09	0.6567
Glutamic	15.3 ± 0.1	15.5 ± 0.1	15.6 ± 0.2	15.5 ± 0.1	15.5 ± 0.1	15.6 ± 0.0	0.6694
Glycine	5.36 ± 0.05	5.09 ± 0.05	5.12 ± 0.21	5.40 ± 0.18	5.18 ± 0.11	5.18 ± 0.18	0.5891
Proline	3.15 ± 0.25	3.11 ± 0.12	2.78 ± 0.09	3.05 ± 0.24	3.19 ± 0.14	3.28 ± 0.09	0.4502
Serine	3.84 ± 0.01	3.78 ± 0.03	3.77 ± 0.02	3.85 ± 0.05	3.83 ± 0.03	3.78 ± 0.05	0.4831
Tyrosine	3.31 ± 0.04	3.37 ± 0.07	3.30 ± 0.05	3.40 ± 0.05	3.30 ± 0.04	3.39 ± 0.03	0.4747

Values are means of triplicate groups and presented as mean ± SE (*n* = 3). Rows without superscript letters were not significantly different among treatments (*p* > 0.05).

**Table 6:** Hematological, plasma biochemical, and antioxidant parameters of hybrid grouper fed the experimental diets at the end of the 11-week feeding trial.

Parameters	Experimental Diets						*p*-Value
	P40	P45	P50	P55	P60	P65	
Hct (%) ^1^	19.8 ± 2.5	21.0 ± 2.6	27.0 ± 5.0	20.6 ± 1.6	25.1 ± 4.3	19.7 ± 3.6	0.5949
Hb (g/dL) ^2^	6.30 ± 0.71	6.97 ± 1.09	7.87 ± 0.83	6.21 ± 0.60	5.62 ± 0.71	6.98 ± 0.58	0.4118
GLU (mg/dL) ^3^	57.3 ± 18.7	31.3 ± 7.4	35.3 ± 3.5	47.3 ± 5.8	44.3 ± 7.4	59.0 ± 13.4	0.4011
TP (g/dL) ^4^	4.90 ± 2.58	5.50 ± 0.87	6.00 ± 0.61	5.37 ± 1.12	4.98 ± 0.69	6.77 ± 1.16	0.9068
TCHO (mg/dL) ^5^	83.5 ± 25.5	123 ± 10	150 ± 20	169 ± 46	183 ± 32	268 ± 89	0.1515
BUN (mg/dL) ^6^	30.2 ± 0.9	32.6 ± 1.1	39.4 ± 5.5	35.2 ± 6.7	31.2 ± 4.9	30.1 ± 2.3	0.6097
GSH (μM) ^7^	30.5 ± 1.6	25.5 ± 3.8	26.5 ± 5.3	29.1 ± 0.4	28.4 ± 0.9	33.2 ± 0.2	0.4540
CAT (nmol/min/mL) ^8^	7.13 ± 2.15 ^b^	5.84 ± 0.70 ^b^	3.61 ± 0.67 ^b^	16.7 ± 3.3 ^a^	7.00 ± 0.90 ^b^	10.5 ± 2.4 ^ab^	0.0072

Values are means of triplicate groups and presented as mean ± SE. Values in the same row sharing the same superscript letter are not significantly different (*p* > 0.05). Rows without superscript letters were not significantly different among treatments (*p* > 0.05). ^1^ Hematocrit; ^2^ Hemoglobin; ^3^ Glucose; ^4^ Total protein; ^5^ Total cholesterol; ^6^ Blood urea nitrogen; ^7^ Glutathione; ^8^ Catalase.

## Data Availability

The original contributions presented in this study are included in the article. Further inquiries can be directed to the corresponding authors.
